# Integrated quantitative proteomic and transcriptomic analysis of lung tumor and control tissue: a lung cancer showcase

**DOI:** 10.18632/oncotarget.7562

**Published:** 2016-02-22

**Authors:** Stefan Tenzer, Petra Leidinger, Christina Backes, Hanno Huwer, Andreas Hildebrandt, Hans-Peter Lenhof, Tanja Wesse, Andre Franke, Eckart Meese, Andreas Keller

**Affiliations:** ^1^ Institute for Immunology, University Medical Center of the Johannes Gutenberg University of Mainz, Mainz, Germany; ^2^ Department of Human Genetics, Saarland University, Homburg, Germany; ^3^ Clinical Bioinformatics, Saarland University, Saarbrücken, Germany; ^4^ SHG Clinics, Völklingen, Germany; ^5^ Software Engineering and Bioinformatics, Johannes Gutenberg University of Mainz, Mainz, Germany; ^6^ Center for Bioinformatics, Saarland University, Saarbrücken, Germany; ^7^ Kiel University, Kiel, Germany

**Keywords:** mass spectrometry, proteomics analysis, transcriptomics, lung tumors, adenocarcinoma

## Abstract

Proteomics analysis of paired cancer and control tissue can be applied to investigate pathological processes in tumors. Advancements in data-independent acquisition mass spectrometry allow for highly reproducible quantitative analysis of complex proteomic patterns. Optimized sample preparation workflows enable integrative multi-omics studies from the same tissue specimens.

We performed ion mobility enhanced, data-independent acquisition MS to characterize the proteome of 21 lung tumor tissues including adenocarcinoma and squamous cell carcinoma (SCC) as compared to control lung tissues of the same patient each. Transcriptomic data were generated for the same specimens. The quantitative proteomic patterns and mRNA abundances were subsequently analyzed using systems biology approaches.

We report a significantly (*p* = 0.0001) larger repertoire of proteins in cancer tissues. 12 proteins were higher in all tumor tissues as compared to matching control tissues. Three proteins, CAV1, CAV2, and RAGE, were vice versa higher in all controls. We also identified characteristic SCC and adenocarcinoma protein patterns. Principal Component Analysis provided evidence that not only cancer from control tissue but also tissue from adenocarcinoma and SCC can be differentiated. Transcriptomic levels of key proteins measured from the same matched tissue samples correlated with the observed protein patterns.

The applied study set-up with paired lung tissue specimens of which different omics are measured, is generally suited for an integrated multi-omics analysis.

## INTRODUCTION

Lung cancer is among the leading cause of cancer related deaths worldwide. In the United States alone, almost 225,000 lung cancer diagnoses are estimated per year, causing 159,260 deaths [1]. Squamous cell carcinoma and adenocarcinoma are routinely differentiated by histological means. Tumor pathologic diagnosis is initially based on bronchoscopic biopsy and can differ from the diagnosis of surgically removed specimens. Although patients with squamous cell carcinoma that mostly develops in smokers show a poorer prognosis than adenocarcinoma patients, prognostic differences in histological types are not considered by the latest TNM classification. Differences in transcriptomic and/or proteomic pattern bear the potential to improve the classification in tumor subtypes.

Recent advances in quantitative mass spectrometry (MS) and the integrative analysis of transcript as well as proteomic levels of tumor samples may support the discovery of disease mechanisms in a systematic manner. A substantial part of MS studies follows a case-control study set-up, where two cohorts of different individuals are compared to each other. Discovery of biomarkers can be enhanced by measuring paired samples, including for example study set ups where both case and control tissue from the same individuals are obtained or samples of the same patient are measured over time.

We explored the number and relative abundance of proteins in adenocarcinoma and squamous cell carcinoma and matched control tissue from the same patients using MS. Respective MS based approaches have become an indispensable tool for molecular and cellular biology and have enhanced the understanding of the complex and dynamic nature of proteomes [2, 3]. Besides applications in metabolomics, MS enables the identification and quantification of several hundreds to thousands of proteins from different sample types, culminating in the first draft of the complete human proteome [4, 5]. The afore-mentioned improvements resulted in a paradigm shift from mere protein profiling and identification towards high-throughput protein quantification. Label-free quantification approaches have emerged as the method of choice for larger sample cohorts, with data-independent acquisition approaches gaining popularity [6, 7].

The complex profiles obtained by quantitative proteomic analyses are frequently used for discovering novel biomarkers [8]. Using gel-based and liquid chromatography-mass spectrometry-based proteomics, even the brain proteome of the 5,300 year old copper age mummy “Ötzi”, the Tyrolean Iceman, was successfully characterized [9]. Beyond different tissues, MS has also been applied to investigate body fluids such as serum [10] or bronchoalveolar lavage fluid [11, 12]. A recent study by McArdle and colleagues explored the value of combining different protein discovery platforms for the development of a multiplexed protein biomarker panel using label-free approaches [13].

Here, we focused on NSCLC as the most common type of lung cancer with squamous cell lung carcinoma (SCC, 25–30%) and adenocarcinomas (40%) as the predominant sub-forms. The detailed grading of the analyzed tumors is provided in Table 1. In an initial step, we assessed the technical reproducibility of the employed MS approach for cancer and control tissues in a paired study set up and estimated the effect sizes. In a second stage, we then explored the lung tissue proteomes of 18 pairs of lung tumor tissue and matched normal lung tissue from the same individuals. The tumor type and TNM classification of samples included in our study are presented in Table 1. Extending our proteomic characterization, we also provide evidence that the applied sample preparation workflow is well suited for an integrative transcriptomic and proteomic analysis.

**Table 1:** Patient details

Tumor Type	TNM
Adeno-Ca	T2aN1
Adeno-Ca	T2aN1
Adeno-Ca	T1bN0
Adeno-Ca	T2aN0
Adeno-Ca	T3N0
Adeno-Ca	T2aN0
Adeno-Ca	T2aN1
Adeno-Ca	T3N1
Adeno-Ca	T3N2
SCC	T1aN0
SCC	T1bN0
SCC	T2bN0
SCC	T2aN0
SCC	T2bN1
SCC	T3N0
SCC	T2aN1
SCC	T3N0
SCC	T4N2

## RESULTS

### Technical reproducibility and variations between individuals in ion mobility enhanced MS

**Figure 1:**
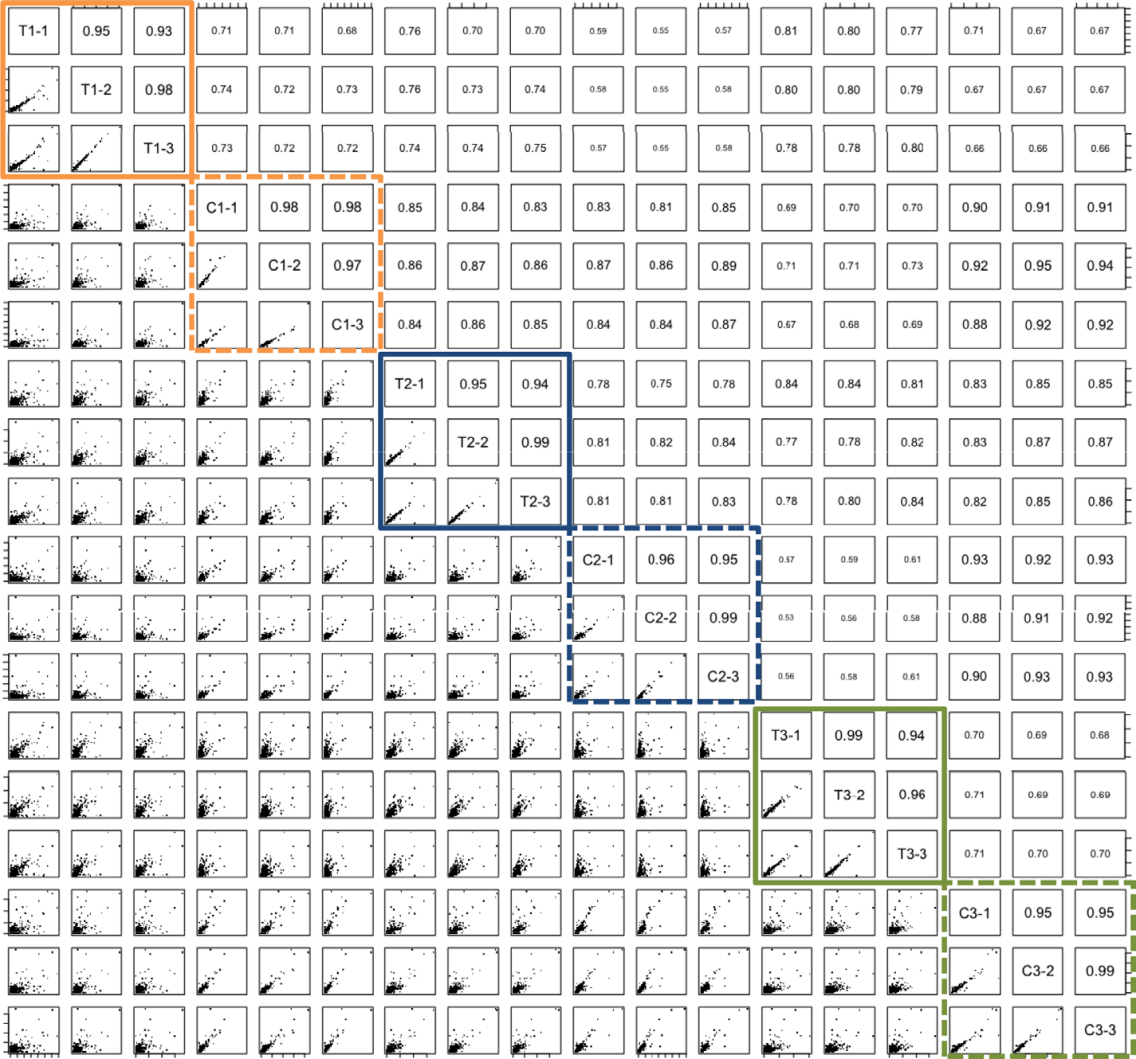
Pair-wise correlation of three tumor (T1-T3) / tissue pairs (C1-C3) that have been measured in technical triplicates Triplicates of tumors are enclosed in solid boxes and of control tissue in dashed boxes. Upper triangle matrix presents the Pearson correlation coefficient, lower triangle matrix thumbnail scatter plots.

For the discovery of protein-based biomarkers, especially for complex marker patterns, a high technical reproducibility of the proteomic workflow is essential. We first explored how accurately technical replicates for paired lung cancer and control tissue can be generated. Three pairs of tumor and respective control tissue were measured in technical triplicates. For the 18 proteome datasets between 1,845 and 2,366 proteins were identified (mean: 2,175; standard deviation: 146). We calculated all 18 × 17/2 = 153 pair-wise Pearson correlations. The respective correlation matrix is presented in Figure 1 where the upper triangle matrix shows the correlation coefficient and the lower triangle matrix contains thumbnails of scatter plots. Most important for this initial analysis are the technical replicates, highlighted for the three individuals in orange, blue and green. The solid rectangles correspond to tumor samples, the dashed ones belong to the paired control tissues. On average we achieved Pearson correlation of 0.96 for technical replicates at standard deviation of 0.02. All other Pearson correlation coefficients were on average 0.77 with standard deviation of 0.10. The set up of the proof-of-concept measurements allowed us to estimate different other group similarities: we can split the values in the correlation matrix in six groups: (tumor / tumor) pairs of the same individual, (control / control) pairs of the same individual, (tumor / control) pairs of the same individual, (tumor / tumor) pairs of different individuals, (control / control) pairs of different individuals and (tumor / control) pairs of different individuals. Only the first two groups represent technical replicates. The average Pearson correlation of each group is presented in Supplementary Figure 1. Best Pearson correlation was obtained for technical replicates of control tissue (0.97) followed by technical replicates of tumor tissue (0.96). The third highest value was reached for control / control measurements of different individuals (0.88). For tumor / tumor replicates of different individuals, average correlation dropped to 0.78. These findings indicate a higher inter-individual heterogeneity in cancer tissue compared to normal lung tissue. For tumor/control correlation of tissue specimen derived from the same individual, we calculated an average value of 0.74 and the lowest correlation was reached for tumor /control pairs derived from different individuals (0.70).

Based on these results we carried out a power calculation. According to the preliminary data we observed effect sizes exceeding 1. Assuming a matched analysis study set up and aiming at an alpha error probability of 0.01 and a statistical power (1- beta error probability) of 0.9 we calculated an approximate cohort size of 18 paired individuals. We thus processed further 9 SCC and 9 adenocarcinoma tissue biopsies and normal healthy lung tissue from the same individual. All subsequent analyses have been carried out on the second cohort of samples.

### Number of proteins per tissue

First, we asked how many different proteins were discovered per sample. On average, we observed 2,661 human proteins per sample (standard deviation of 187 proteins), ranging from 2,090 up to 2,916 proteins. In total, 3,328 different proteins were identified across all 18 tissue pairs (36 samples) analyzed. These are presented in Supplementary Table 1 along with selected performance characteristics of the respective features. By comparing the number of detectable proteins in cancer tissue relative to the matched control tissue we discovered an increased complexity of the proteome in the cancer tissue (average protein number in cancer tissue: 2,783, average protein number in control tissue: 2,540). Supplementary Figure 2 shows a scatter plot, where each data point represents a pair of tumor / control tissue. In one of 18 cases the numbers for both tissues matched well, in one case the control tissue showed more proteins and in 16 cases the tumor tissue had a larger repertoire of proteins, which may be indicative of tissue dedifferentiation. A two-tailed paired *t*-test indicated that the differences between both tissues were significant (*p* = 0.0001). Between adenocarcinoma and SCC we did not found significantly changed alterations in the number of detectable proteins.

In a similar fashion we investigated how many proteins were detected in the four groups of adenocarcinoma, controls of adenocarcinoma, SCC, and controls of SCC. The result is provided as Venn diagram in Supplementary Figure 3. In brief, 2,824 proteins were present in all tissue groups. 37 proteins were only found in adenocarcinoma tissue, 10 only in SCC, 6 and 3 in the respective controls. An additional 73 proteins were discovered only in adenocarcinoma and SCC tissue while not in controls.

### Quantitative analysis of protein abundance in tumors and controls

Since already the proteome diversity varied significantly between cancer and control tissue we asked whether specific proteins are higher or lower abundant in tumor relative to control tissue. Paired two-tailed *t*-tests that have been carried out for each protein separately and adjusted for multiple testing using Benjamini-Hochberg adjustment. This analysis revealed as many as 1,736 proteins with adjusted *p*-values below 0.05. Of these, 404 (23%) were higher abundant in control tissue while 1,332 proteins (77%) showed higher abundance in cancer tissue. Three proteins, CAV1, CAV2, and RAGE, were higher in all control tissues as compared to corresponding cancer tissues. Vice versa, 12 proteins were higher in all tumor tissues as compared to the corresponding cancer tissues (ROA1, ELOB, ALDOA, PP14B, HNRPC, RLA2, SRSF9, CALU, SC61B, FHL2, G3BP1 and PABP4). The protein showing highest up-regulation in tumor tissue was HEM6 with raw and adjusted *p*-value of 6 × 10^−10^ and 1.3 × 10^−8^. The other extreme, being most significantly down-regulated in lung cancer, was CATA with raw and adjusted *p*-value of 7.7 × 10^−10^ and 1.3 × 10^−8^. The top-20 proteins with respect to adjusted *t*-test significance values are represented graphically as heat map in Figure 2A. In this heat map all tumor samples cluster at the right hand side and all controls at the left hand side. As described in the Methods section we tested different values for the k most variable proteins. In general, this parameter had a limited influence, as Figure 2B demonstrates. Here, the top 50 proteins are presented, and still an almost perfect clustering in both groups is obtained. The expression of each protein in both groups with raw and adjusted *p*-values and the area under the receiver operator characteristics curve are presented in Supplementary Table 2.

**Figure 2A:**
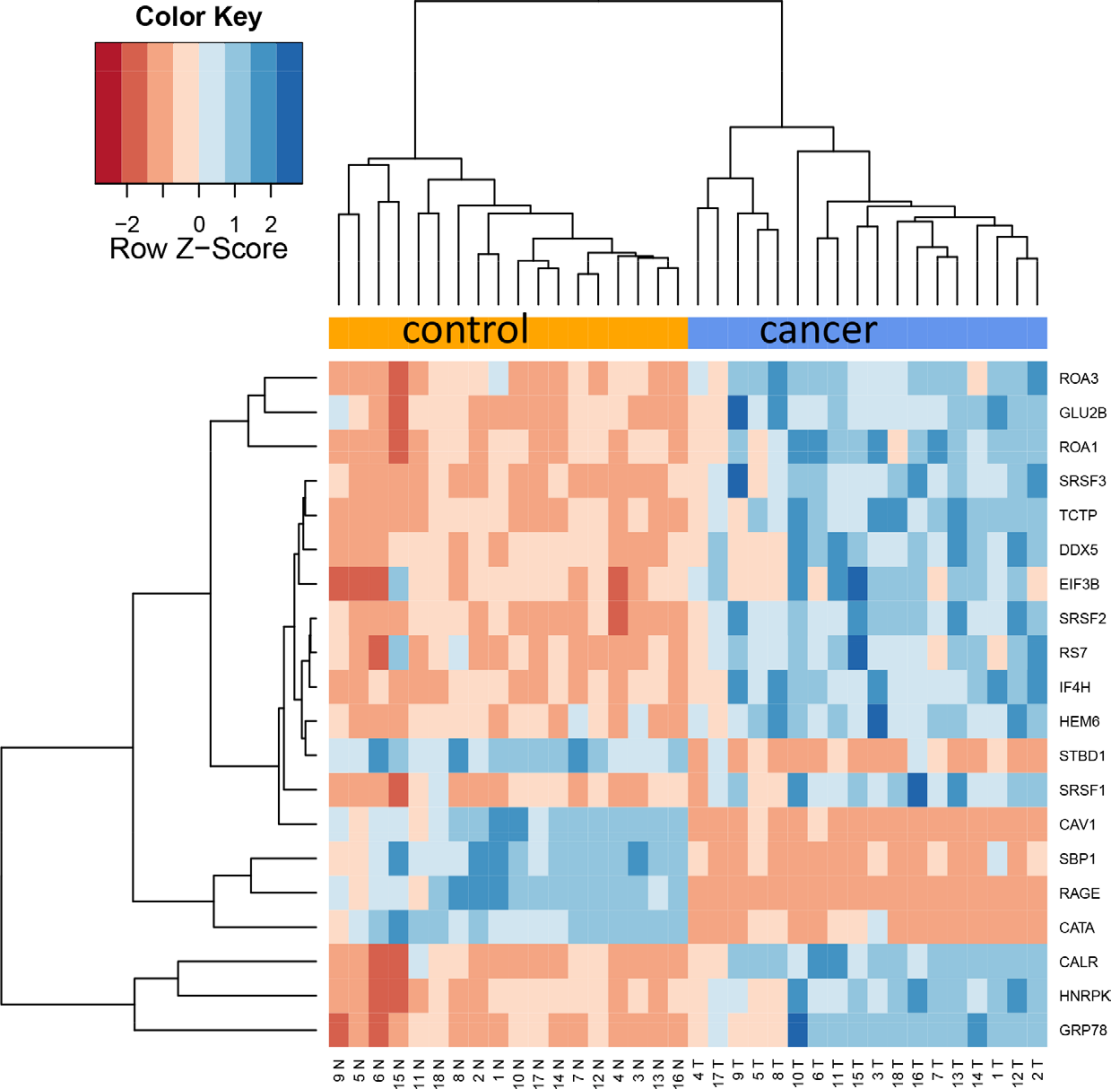
Cluster heat map of 20 most differentially abundant proteins The heat map shows a clear separation of lung cancer samples on the right hand side and control samples on the left side.

**Figure 2B:**
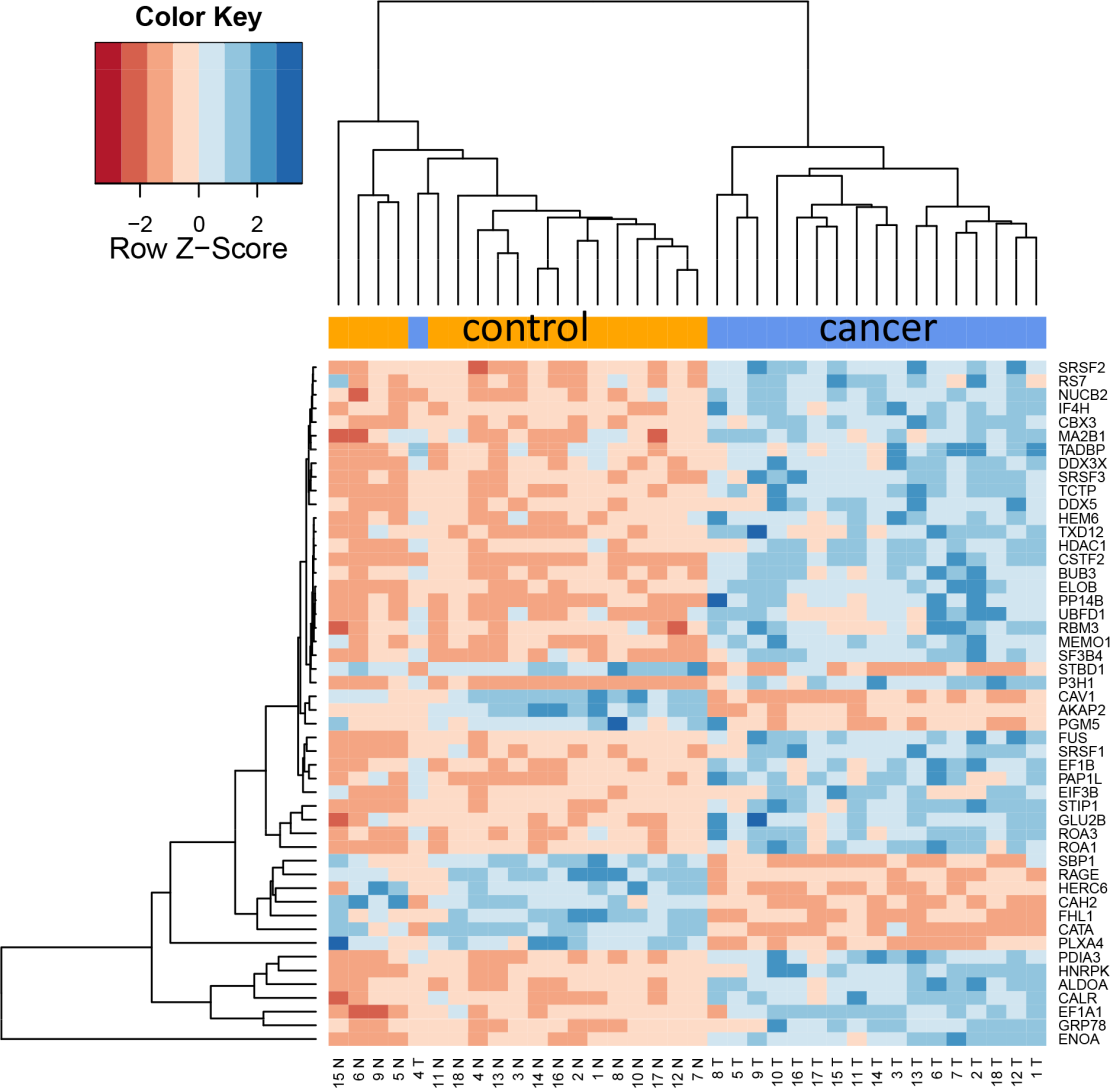
Cluster heat map of 50 most differentially abundant proteins Only one cancer sample clusters with the controls.

### Differences in the mRNA levels of corresponding genes

**Figure 3:**
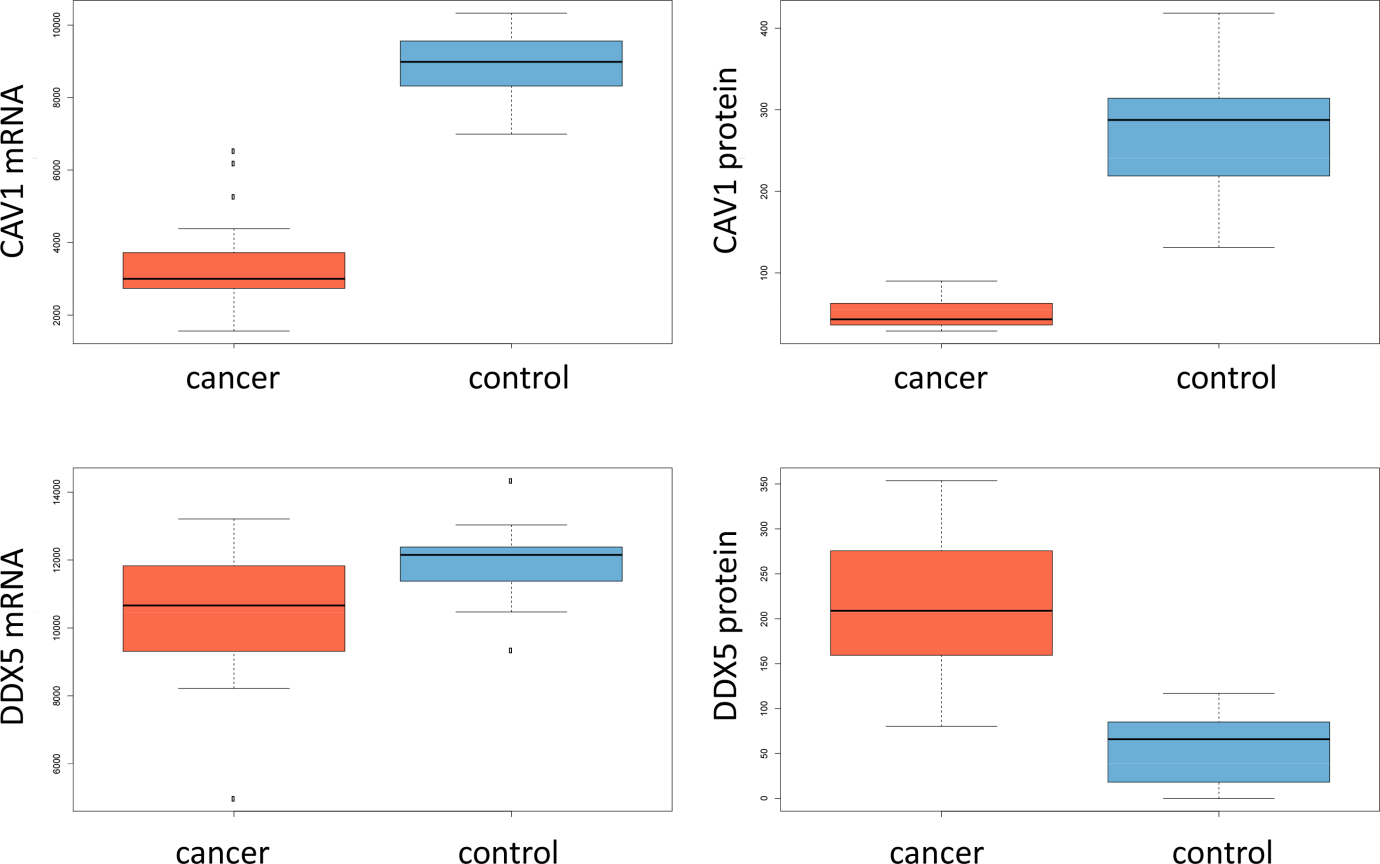
Box-plots for mRNA expression and protein abundance in cancer and controls for CAV1 and DDX5

Since one aim of our study was to investigate the gene expression level of transcripts from the same biopsies we investigated expression patterns of genes encoding for the 20 proteins from Figure 2A described in the last paragraph. The set contains 5 proteins with lower abundance in cancer. For all of them we found significantly lower gene expression in cancer, confirming the proteomic data on transcriptome level. Four of them were significant after adjustment for multiple testing with *p*-values of 7 × 10^−6^ or lower. For the remaining 15 proteins with higher expression levels in cancer, we could observed elevated transcript levels in 11 cases. Although the transcriptomic measurements could be used generally as surrogate for the protein measurements due to the high concordance we also found counterexamples. An interesting case is the gene/protein DDX5. This gene is significantly down-regulated in tumor tissues on mRNA level but significantly up-regulated on the protein level. The same holds for SFRS3. The best matching example, CAV1, as well as DDX5 are presented as box plots for proteomic and transcriptomic expression in Figure 3. Table 2 summarizes the normalized mRNA expression values for the 20 proteins together with the direction of regulation.

**Table 2:** Comparison of mRNA and protein abundance of selected genes/proteins

Gene	Protein	Protein Deregulation in cancer	median mRNA Control	median mRNA Cancer	*t*-Test raw *p*-Value	*t*-Test adjusted *p*-Value	AUC
CAT	CATA	down	6880	2978	1.24E-09	4.40E-07	0.02
CAV1	CAV1	down	8984	2999	1.87E-10	1.27E-07	0.00
AGER	RAGE	down	3165	242	6.92E-08	6.98E-06	0.01
SELENBP1	SBP1	down	4319	739	3.52E-09	8.53E-07	0.00
STBD1	STBD1	down	351	282	9.48E-01	9.74E-01	0.28
CALR	CALR	up	1176	1689	7.20E-05	1.12E-03	0.84
DDX5	DDX5	up	12153	10661	9.90E-03	4.48E-02	0.21
EIF3B	EIF3B	up	2769	3903	9.46E-05	1.37E-03	0.89
PRKCSH	GLU2B	up	754	1039	8.31E-05	1.24E-03	0.87
HSPA5	GRP78	up	1894	1490	4.18E-02	1.30E-01	0.28
CPOX	HEM6	up	1312	1947	1.24E-04	1.67E-03	0.87
HNRNPK	HNRPK	up	2353	2537	8.12E-02	2.09E-01	0.65
EIF4H	IF4H	up	5356	5592	8.36E-02	2.14E-01	0.61
HNRNPA1	ROA1	up	211	292	1.52E-03	1.09E-02	0.80
HNRNPA3	ROA3	up	594	778	6.89E-04	6.01E-03	0.92
RPS7	RS7	up	348	424	1.04E-03	8.22E-03	0.81
SFRS1	SRSF1	up	4697	5513	3.83E-02	1.22E-01	0.76
SFRS2	SRSF2	up	6500	6947	7.33E-02	1.94E-01	0.69
SFRS3	SRSF3	up	1154	923	8.29E-04	6.93E-03	0.20
TPT1	TCTP	up	10058	9029	2.12E-02	7.86E-02	0.29

### Differences in proteomics patterns of different grades

We also compared the protein pattern in T1 and T2 tumors versus the abundance in T3 versus T4 tumors (details on the T stages are provided in Table 1). Although several proteins showed substantial fold changes, none of them remained significant after adjustment for multiple testing, which may however be due to the limited cohort size for this comparison. Among the most altered proteins were HNRPL and RBBP4 (*p* = 0.0002 and *p* = 0.0004, respectively). Interestingly, we observed a generally lower protein abundance in T3 and T4 tumors as compared to T1 and T2 tumors. Among the top 20 proteins none was more abundant in T3 or T4 tumors. Especially three proteins were not detected in T3 and T4 proteins while we found hits in T1 and T2 tumors (CPSF7, IF1AY and CJ118).

### Differences in protein abundance between adenocarcinoma and SCC

**Figure 4:**
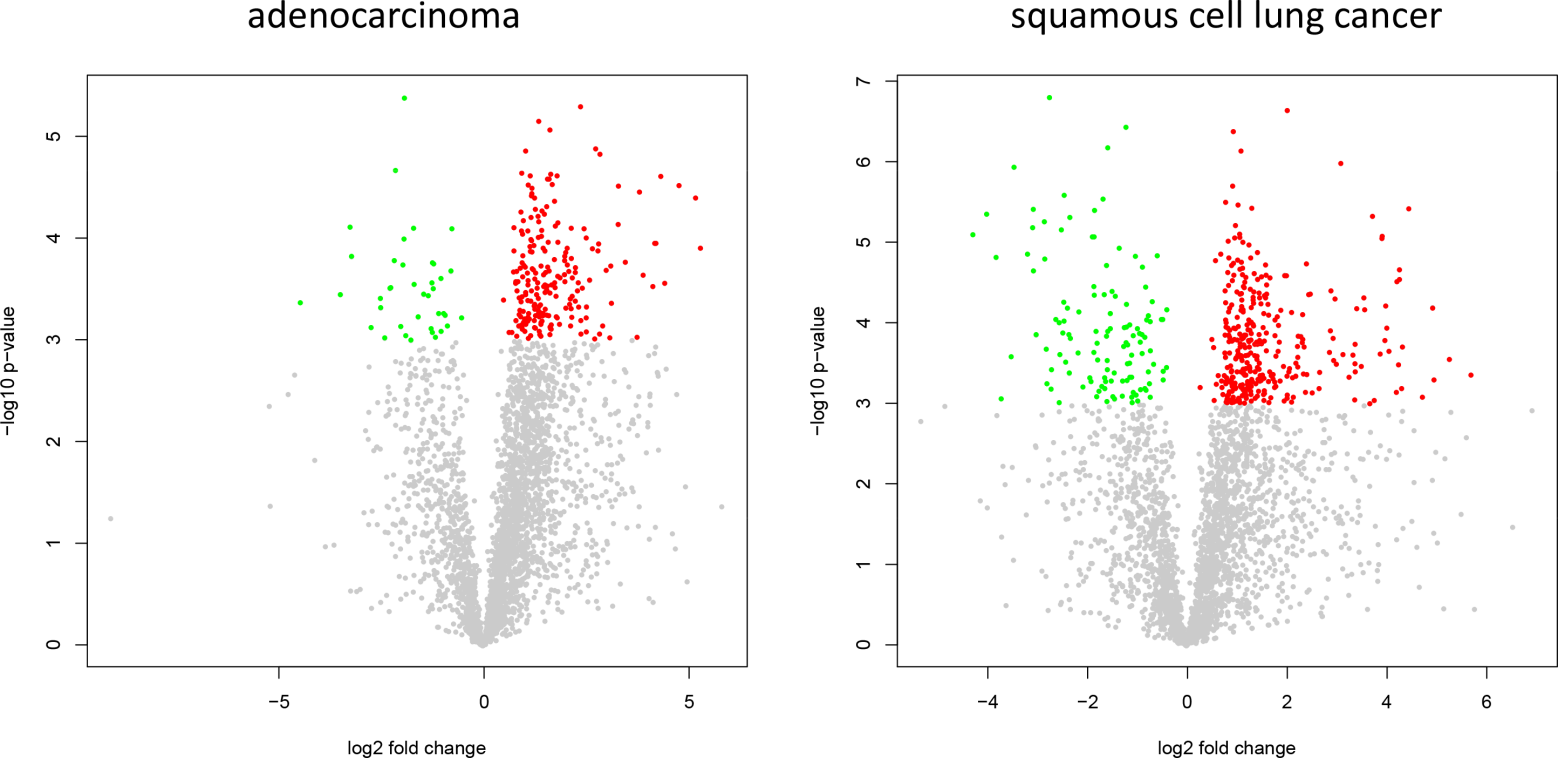
Volcano plots for adenocarcinoma and SCC The plots present log_2_ of the fold change versus negative decimal logarithm of the *p*-value. Red data points correspond to up-regulated and green data points to down regulated proteins.

**Figure 5:**
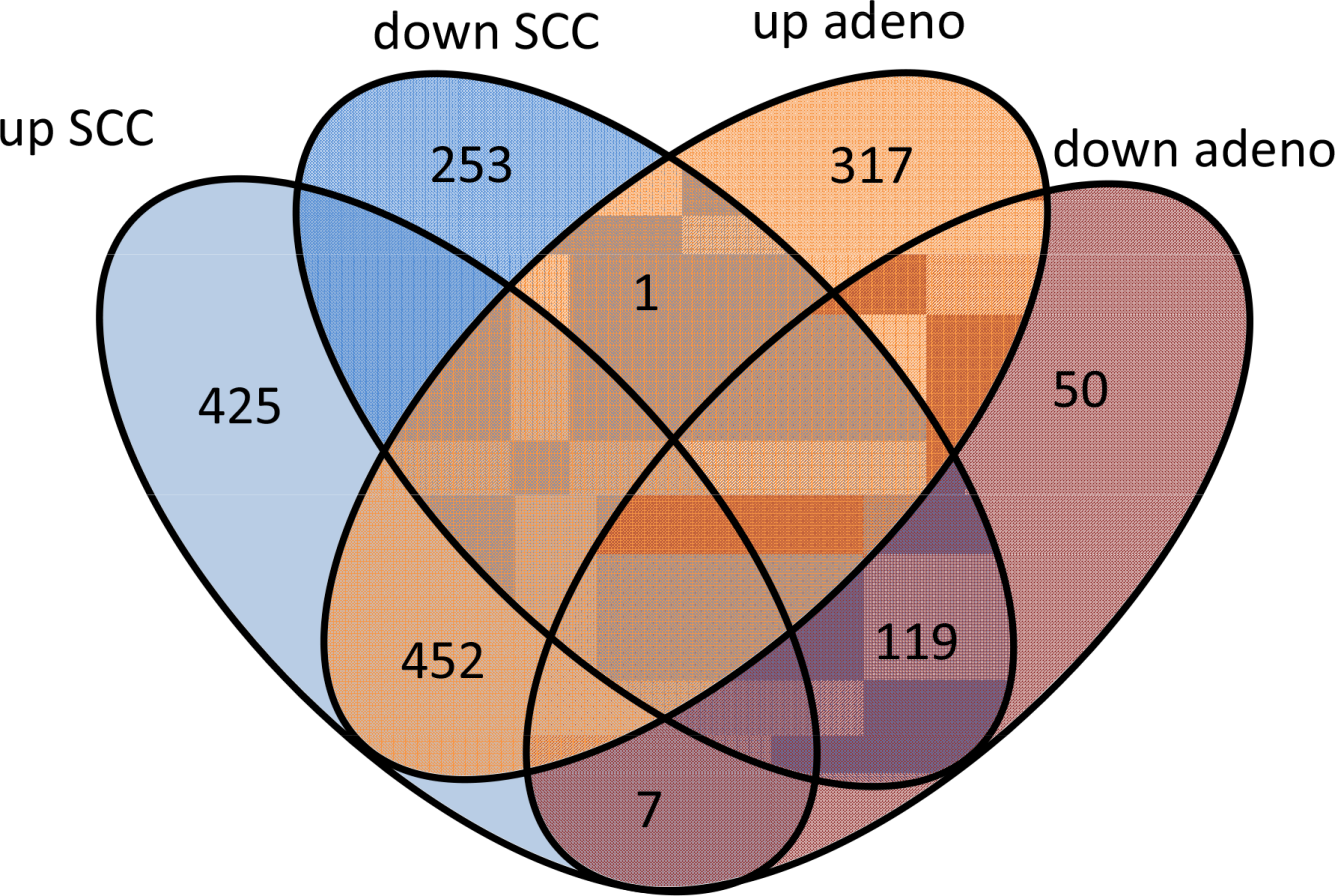
Venn diagram of proteins that are up and down-regulated in SCC and adenocarcinoma Seven proteins were up-regulated in SCC and down in adenocarcinoma. One protein was down-regulated in SCC but up-regulated in adenocarcinoma.

In comparing adenocarcinoma and squamous cell lung cancer proteomes we observed 37 proteins only present in adenocarcinoma and 10 only present in SCC. The volcano plots in Figure 4, showing the fold change (log base 2) versus the negative decimal logarithm of the significance value for the paired comparisons demonstrate a different behavior. For adenocarcinoma, the majority of proteins was higher abundant in cancer tissue compared to controls (red data points in Figure 4). For SCC, proteins more equally scatter between up- and down-regulated markers, but still the protein levels are overall higher in cancer samples. Figure 5 compares up- and down-regulated proteins in adenocarcinoma and SCC in a Venn diagram. 452 proteins were up-regulated in SCC and adenocarcinoma, 119 were down-regulated in both types of lung cancer. We however also observed one protein that was down-regulated in SCC while up-regulated in adenocarcinoma (VWA8) and vice versa 7 proteins showing the opposite behavior (FAK2, PMM2, K2C5, SMCA5, FAD1, DTX3L and CLU). To further support the hypothesis that not only cancer tissue differs from control tissue but also differences between SCC and adenocarcinoma exist, we performed unsupervised hierarchical clustering of the 50 most variable proteins and principal component analysis. The result of the clustering is presented in Figure 6A as heat map with dendrograms on top (samples) and left (proteins). As dendrogram and heat map clearly demonstrate, adenocarcinoma and controls from adenocarcinoma patients have a tendency to cluster together. While all adenocarcinoma samples cluster perfectly, the respective controls scatter on the left and right hand side. Besides one SCC control sample that matches to the adenocarcinoma controls, we observed a perfect clustering in adenocarcinoma patients and SCC patients. The SCC samples and the respective control tissues showed a higher proximity to each other, with clear distinction between both groups. Only one control sample clustered with the SCC tissues. To determine the number of clusters we applied different scores relying e.g. on sum-of-squares clustering or the Silhouette Score. We found an optimal number of 2 clusters in the samples. The average Silhouette Scores were 0.53 and 0.47, respectively. Scores for all samples are presented in Supplementary Figure 4. The clustering also highlighted a set of proteins that was almost solely present in control tissue of adenocarcinoma patients, including FANCJ, CCAR2, AGRIN, and LAMC1 (Figure 6A). Since the results of the clustering are based on the 50 most variable proteins, we also generated a 2D scatterplot of the high-dimensional proteomics patterns using principal component analysis (PCA). In Figure 6B, the first versus the second principal component are presented. Importantly, the first principal component almost perfectly separates control tissues on the right hand side from cancer biopsies on the left. Focusing on the second principal component we can detect that here SCC and matched SCC control tissue are located on top while adenocarcinoma and matched controls are located on bottom of the scatter plot. Control tissues have generally a lower variance as compared to cancer tissues and are clustered closer to each other as compared to the cancer tissues. In sum we can distinguish the four groups in the principal component analysis: adenocarcinoma and adenocarcinoma control tissue as well as SCC and SCC control tissue.

**Figure 6A:**
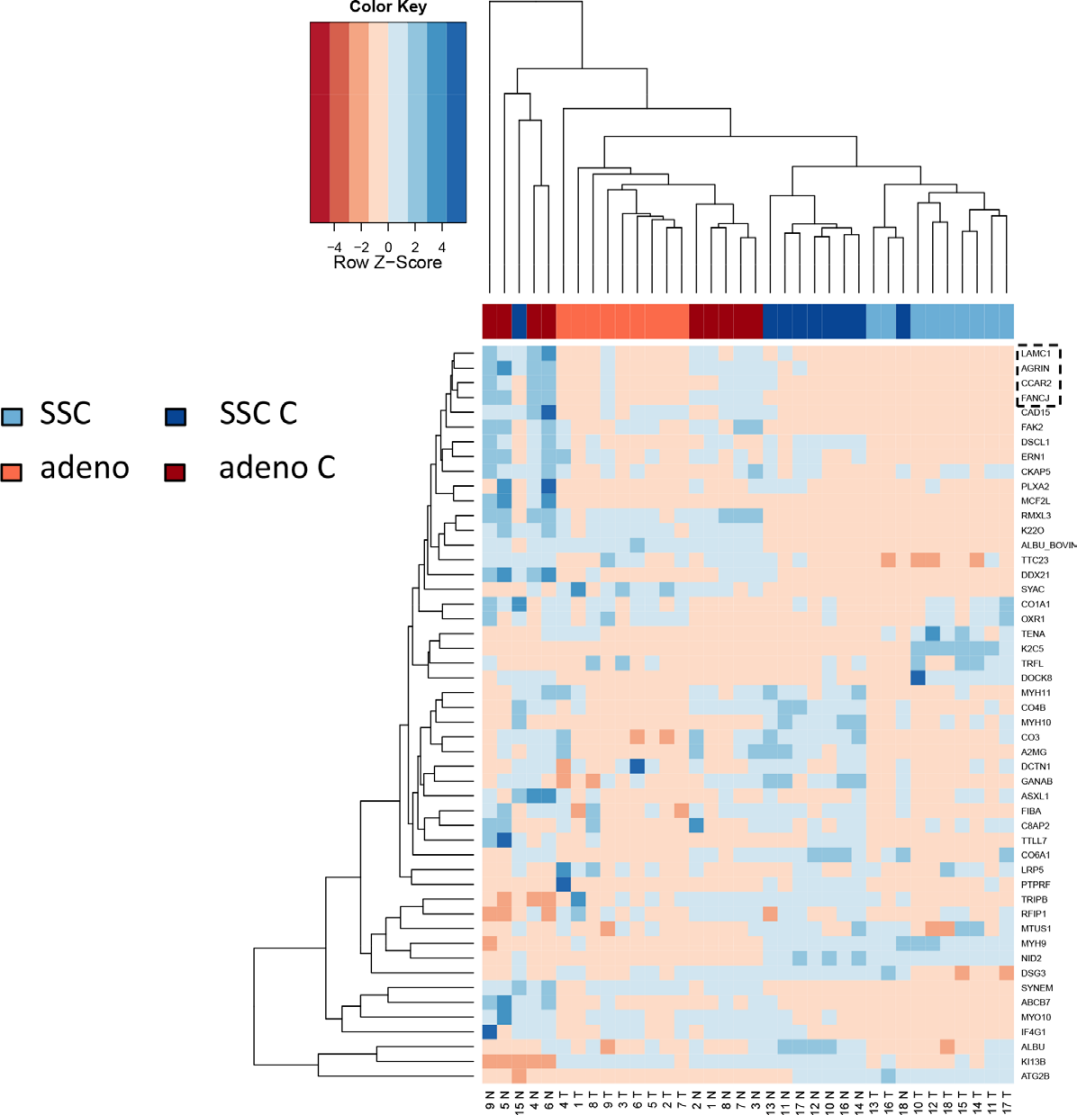
Unsupervised cluster analysis of the 50 most variable proteins for the four groups (adenocarcinoma and paired controls, SCCand paired controls) The dendrogram indicates that SCC and adenocarcinoma tend to cluster together. Inside of both clusters, cancer tissue clusters apart from control tissue. Especially four proteins that are solely expressed in adenocarcinoma samples are exemplarily highlighted by a dashed box.

**Figure 6B:**
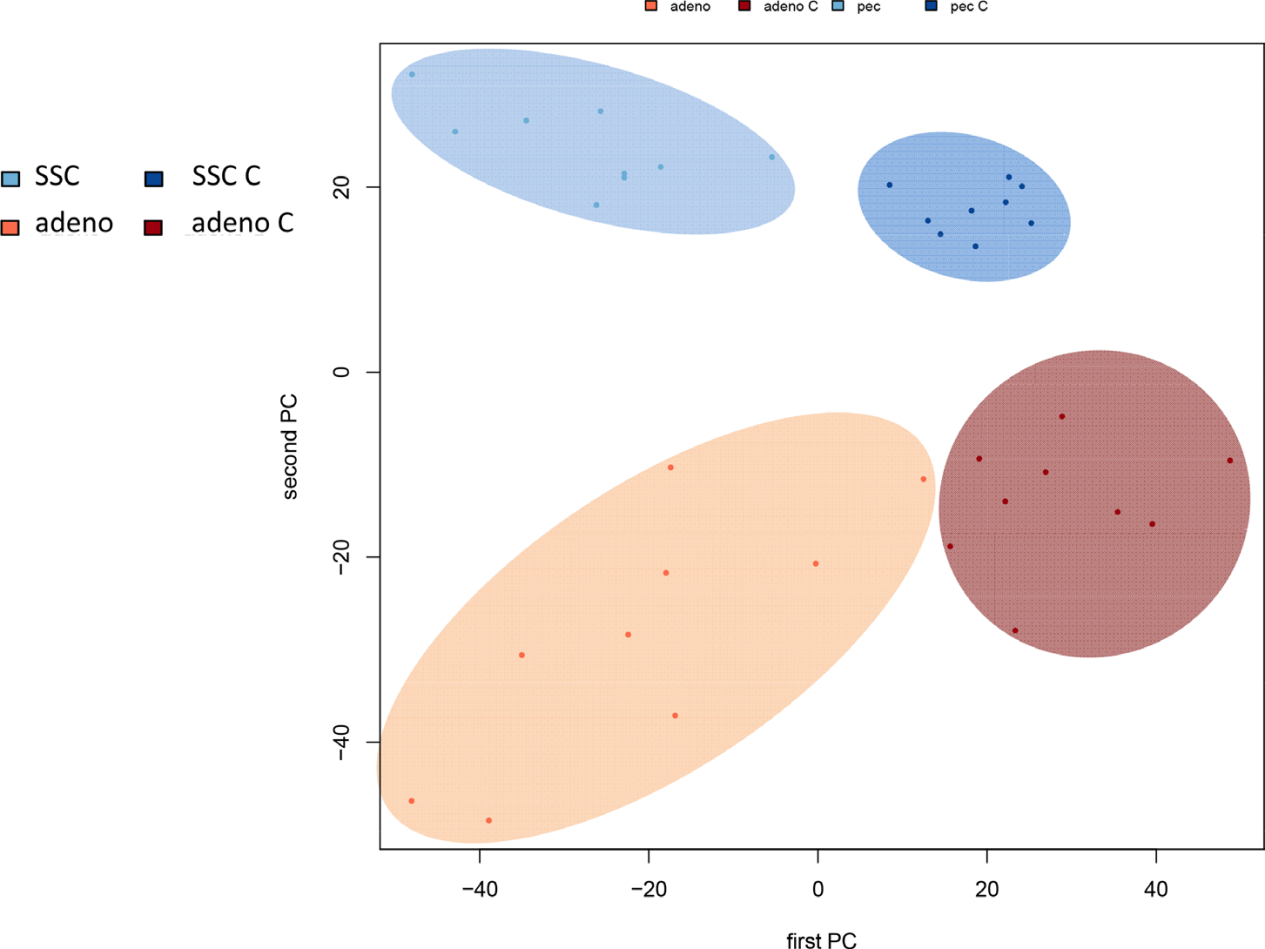
Principal component analysis of all samples represented by a scatter plot of the first versus second principal component The first PC distinguishes cancer from controls, the second PC adenocarcinoma from SCC.

The comparison of adenocarcinoma and SCC requires an unpaired analysis. On proteome level, we observed 17 differentially expressed proteins, which remained significant following adjustment for multiple testing. Of these, 10 were more abundant in adenocarcinoma tissues (AAKB1, ACPM, FA49A, K1C9, MYL9, PPM1F, RMXL3, SH3L1, SYAP1 and PAPS2). The remaining 7 were measured at a higher amount in SCC tissue biopsies (ATG2B, CTNA3, NID2, RBBP7, SF3B1, TNPO1, UBA1). As for the general lung cancer proteins presented above we asked for the expression of genes that are translated into the respective proteins. While we found significantly aberrant expression in this previous case we here generally observed non-significant differential regulation of mRNAs. For one up- and down-regulated protein we obtained significant *p*-values prior to multiple hypothesis testing adjustment on mRNA level. RBBP7, which was down-regulated on proteome level in adenocarcinoma had also significantly lower gene expression in adenocarcinoma tissues. PAPS2, which was higher abundant in adenocarcinoma was likewise significantly up-regulated in the transcriptome of adenocarcinoma tissues. For the remaining proteins the direction of regulation frequently matched, however, significance values were above the alpha level. One examples is MYL9, which is higher expressed in adenocarcinoma on transcriptomic and proteomic level, but while the protein expression was significantly different, the mRNA significance value not significant (*p* = 0.1). For RBBP7 the gene and protein expression compared to each other in the four groups (adenocarcinoma, adenocarcinoma control, SCC and SCC control tissue) is presented in Figure 6C.

**Figure 6C:**
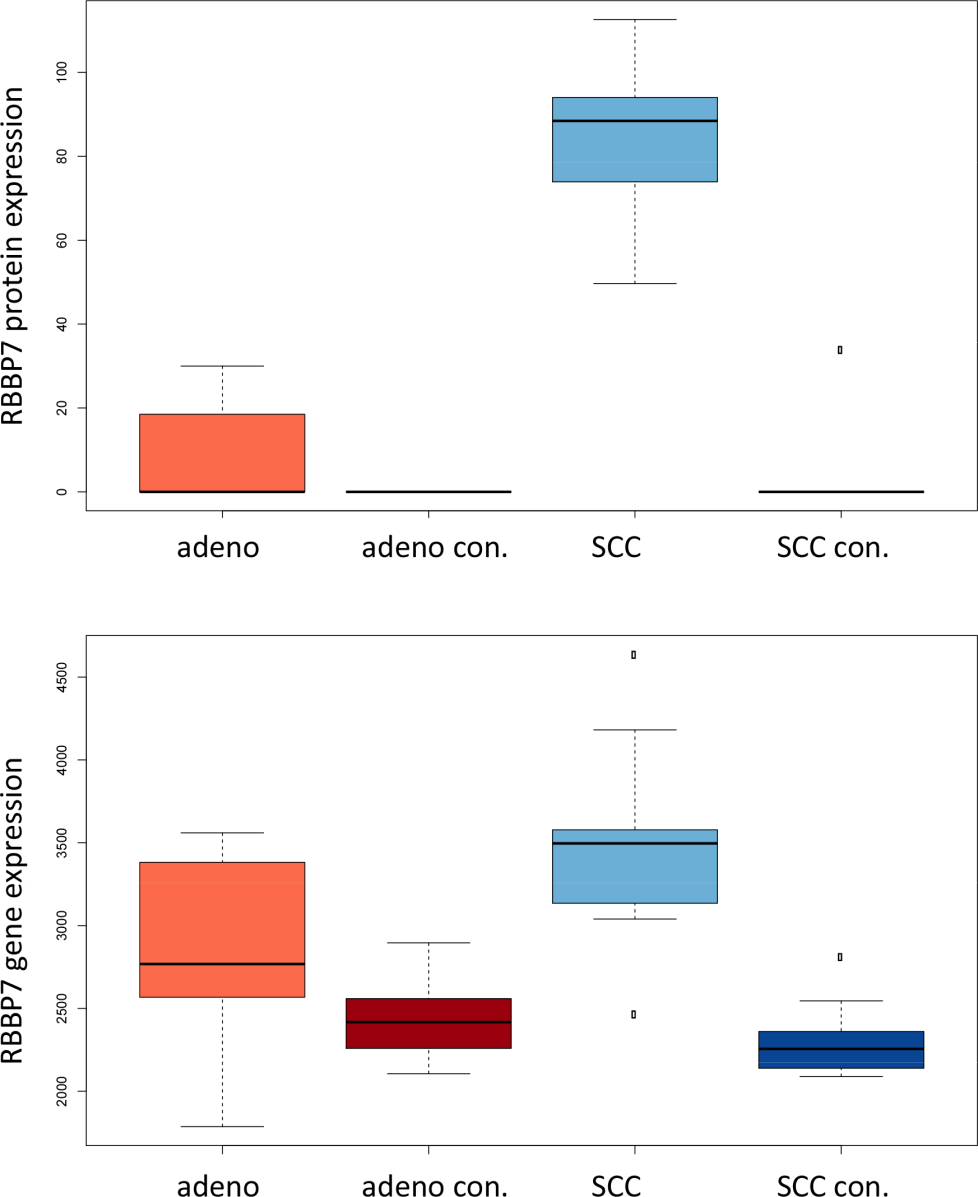
Expression of RBB7 in adenocarcinoma, SCC and respective controls in mRNA and proteins are presented For mRNA and protein expression, SCC have the highest levels among the four groups.

## DISCUSSION

In the present study we investigated the proteome from paired cancer and control samples and matched mRNA to protein abundance.

Analyzing paired lung cancer and control lung tissues we observed an increased repertoire of proteins in cancer tissues as compared to the matched controls. It remains to be seen if and to what extend the increased complexity of the proteome is related to the process of de-differentiation of the highly specialized lung epithelium during carcinogenesis. Transcriptomic alterations that occur during lung cancer development include over-expressed cell cycle related genes like E2F3, BUB3, CDK4, MCM2, MCM3, and MCM7 as summarized by Powell and Borczuk [14]. Analyzing the abundance for the respective proteins, we observed a significant over-expression (adjusted *t*-test *p*-values of the proteins: BUB3 – 4 × 10^−6^; MCM2 – 0.004; MCM7 – 0.004; MCM3 – 0.007). MCM6 was found to be over-expressed with a *p*-value of 0.014. For NTF2, which is known to be increased during the transition from adenoma to carcinoma, we observed an up-regulation of the protein but only with a *p*-value of 0.06. Although smoking has been described as important confounding variable for gene expression in lung cancer [15]. we did not include the smoking status in our study due to the lack of comprehensive information on the smoking history of our patients.

Interpreting the profiles we observed also differences in controls of adenocarcinoma as well as SCC controls. Besides biological reasons, batch effects could also confound the pattern. We did however not found different complexity of the proteome of adenocarcinoma and SCC patients, arguing against a respective bias. These differences may again be due to effects in the measurement procedure. Also pre-malignant changes in tissue could be a reasonable source for the observed variability [16].

The most up- or down-regulated proteins in this study are correlated to lung cancer in the literature. Reduced SBP1 levels are correlated to poor outcome in adenocarcinoma [17]. CAV1 is lower expressed in lung adenocarcinomas and has likewise prognostic potential [18]. The anti-apoptotic protein TCTP is known to be up-regulated in lung cancer cells [19]. GRP78 is known as key player in lung cancer development and progression [20, 21]. In addition to the de-regulated proteins we also investigated the proteins, which were most equally expressed in our study, i.e. proteins that showed the least difference in their abundance between lung cancer tissues and controls. However, several of these proteins have previously been associated with lung cancer. For example, TIMP3 was reported to be down regulated on the transcriptomic level of pulmonary adenocarcinomas [22]. Likewise, CD59, which our analysis identified with equal abundance in lung cancer and controls, was previously identified as over-expressed in NSCLC [23]. Also ICAM1, which also showed an equal abundance in lung cancer and controls in our study, was previously discussed as important for lung cancer [24]. However, it is also important to indicate that in the aforementioned comparisons between our study and the studies of others, we found concordant results for several proteins including caveolin 1 and caveolin 2, both of which were reported as down-regulated markers in lung cancer [22] and both of which were also among the most substantially down-regulated proteins in our study.

As for the results that are not or not fully concordant between our data and the studies by others there are several possible explanations including i) a lack of comparability (studies are done on cell lines versus studies on tissues, expression analysis is performed on only the transcriptomic or the proteomic level), ii) experimental bias in the protein measurement in our study, and iii) biological variability, especially in the light of the small cohorts typically investigated in high-throughput studies.

For the majority of proteins we calculated likewise significantly de-regulated mRNA levels. Exceptions are DDX5 or SFRS3, where protein and gene expression did not matched. Although it is known that mRNA expression is not always a perfect surrogate for protein abundance [25] aberrations between cancer and control samples may indicate a pathological change in the regulation of the two proteins.

In summary, we measured protein abundance in a stable and reproducible manner indicating that even single protein measurement bears diagnostic potential. We also demonstrate that marker signatures differentiate not between lung cancer and matched control tissue but also between tissue from adenocarcinoma and SCC. Prospective studies with independently measured cohorts will be required to further strengthen the conclusion of our results.

## CONCLUSIONS

We performed an integrative multi-omics study containing proteomics and transcriptomics data from the same tissues. Lung cancer tissue and control lung tissue were obtained from the same individuals, allowing for paired data analysis. Our results indicate that data-independent acquisition workflows can be applied to discover tumor- and/or tissue specific biomarkers. Bioinformatics analyses demonstrate that the quantitative proteome profiles enable to distinguish between lung cancer tissues and matched normal tissues, as well as between different lung cancer subtypes. There is a general change in the proteome of lung tumor tissue as shown by a broader proteome variety in tumor versus control tissues. Cluster analysis revealed tumor-type specific subsets of potential biomarkers. Transcriptomic measurements of the same tissue samples showed a large concordance to the proteomics pattern. The study design is to allow an integrated multi-omics analysis.

## METHODS

### Patients and tissue collection

Tissue specimens were obtained with patient informed consent from SHG clinic, Völklingen, Heart Center during lung cancer resection. The study was approved by the local ethics committee (Ärztekammer des Saarlandes, 01/08). After resection, tissue specimen were subsequently transferred into RNAlater TissueProtect Tubes (Qiagen) and incubated over night at 4°C. The next day, RNAlater solution was removed and the tissue specimen were transferred into −80°C freezer for long-term storage.

In total, we obtained lung cancer and normal healthy lung tissue from 21 patients, including 11 patients suffering from adenocarcinoma and 10 patients suffering from squamous cell lung carcinoma. The patient details are provided in Table 1. In this study, the control tissues and cancer tissues have been obtained in each case from the same patient, allowing for a paired data analysis.

### Quantitative proteomic analyses

To obtain proteins and RNA from the same piece of tissue, the tissue sample was transferred into 1 ml Qiazol lysis reagent and disrupted using 7 mm stainless steel beads for 5 min 50 Hz. Then 200 μl chloroform was added, vigorously vortexed and incubated for 3 min at room temperature. After centrifugation for 15 min, 14.000 rpm and 4°C, the aqueous phase was subsequently used for RNA isolation with the miRNeasy Mini Kit (Qiagen) and the organic phase was stored at −80°C until shipping on dry ice to the Forschungszentrum Immunologie (Mainz, Germany) for protein isolation and quantitative proteomic analyses. Protein containing organic phase fractions of RNA preparations were lyophilized and re-solubilized in lysis buffer (7 M urea, 2 M thiourea, 5 mM DTT, 2% CHAPS) by sonication for 10 min at 4°C. After resolubilization, protein concentrations were determined using a 660 nm assay (Thermo) according to manufacturers instructions. 20 μg of total protein were used for tryptic digestion by applying a modified FASP protocol [26]. After digestion, tryptic peptides were lyophilized and dissolved in 0.1% formic acid and spiked with 20 fmol/μL of yeast enolase 1 MassPREP^™^ protein digestion standard (Waters) prior to LC-MS analysis.

Tryptic peptides (300 ng / injection) were analyzed using a nanoscale UPLC system (nanoAcquityUPLC (Waters)) coupled online to a Synapt G2-S HDMS mass spectrometer (Waters). Peptides were separated on a HSS-T3 1.7 μm, 75 μm × 250 mm reversed-phase column (Waters) using direct injection mode. Water (UPLC grade, Roth) containing 0.1% formic acid (Fisher Scientific) was used as mobile phase A and acetonitrile (UPLC grade, Roth) containing 0.1% formic acid as mobile phase B. Peptides were eluted with a gradient of 5–40% mobile phase B over 90 min at a flow rate of 300 nL/min and a temperature of 55°C. Afterwards, the column was rinsed with 90% mobile phase B for 10 min and re-equilibrated resulting in a total analysis time of 120 min. Analysis was performed in positive mode ESI-MS using MS^E^ in combination with on-line ion-mobility separation (UDMS^E^) as described in detail by Distler et al. [26]. The data were post-acquisition lock mass corrected using [Glu1]-Fibrinopeptide B. LC-MS data were processed using ProteinLynxGlobalSERVER version 3.0.2 (PLGS, Waters Corporation) searching against the UniprotKB/Swissprot human database (UniProtKB release 2014_02, 20,266 entries), which was concatenated to a reversed decoy database, using the following search criteria for peptide identification: i) trypsin as digestion enzyme ii) up to two missed cleavages allowed iii) fixed carbamidomethylcysteine and variable methionine oxidation as modifications, iv) minimum three identified fragment ions. Precursor and fragment ion mass tolerances were automatically determined by PLGS3.0.2 during database search, resulting in mass tolerances below 5 ppm (3.3 ppm RMS) for precursor and below 10 ppm for fragment ions. The initial false discovery rate (FDR) for protein identification was set to 1% in PLGS. Data post-processing was performed using the software package ISOQuant, including retention time alignment, exact-mass-retention-time and ion-mobility clustering, signal annotation, normalization and protein isoform/homology filtering as described in [27]. Absolute in-sample amounts were calculated in ISOQuant for each protein based on the TOP3 approach [28].

To minimize potential batch effects we alternated measurements between cancer and control tissue and did not measured batches of cancer and controls.

### mRNA analysis

From the same tissue specimens, RNA has been isolated and transcriptomic patterns have been measured. As profiling technique HumanHT-12 v4 Expression BeadChip Kit (Illumina) has been applied according to manufacturers instructions. Raw data have bee generated by the HiScan system and averaged expression intensity as well as detection *p*-values have been extracted. Prior to further processing quantile normalization has been applied. Gene and protein IDs were matched using publicly available data from GeneCards (http://www.genecards.org/).

### Bioinformatics analysis

In a first stage, we analyzed three pairs of tumor tissue / control tissue in technical triplicates to assess technical variations and to estimate the required cohort size. Here, pair-wise Pearson correlation coefficients were calculated. From these, an a-priori power calculation was carried out to estimate the required cohort size of the actual study. The downstream analysis of proteomics pattern has been performed in R (version 3.0.2). Significance values were calculated by two-tailed paired *t*-tests unless mentioned explicitly. Additionally, the area under the receiver characteristic curve (AUC) was assessed. For the AUC, expression of individual proteins / mRNAs has been considered. The closer AUC values to 0.5 were, the less differentially regulated the respective proteins / mRNAs are. The closer the AUC to 1 the more up-regulated, the closer the AUC to 0 the more down-regulated are the markers. For hierarchical clustering the R “hclust“ function has been applied with the Euclidian distance as distance measure. To calculate the optimal number of clusters in the data the “NbClust” package was used. Silhouette Scores were calculated and visualized with the “cluster” R package. Additionally, different clustering using the k most variables have been tested (k = 10, 20, 50, 100, 150, 200, 250, 500) and representative examples were included in the manuscript. To make protein expression levels comparable to each other, for this analysis the z-score of each proteins abundance level was calculated. Principal component analysis (PCA) has been performed using the R “pca” function. All *p*-values through the study were adjusted for multiple testing using the Benjamini-Hochberg approach if not mentioned explicitly.

## SUPPLEMENTARY MATERIALS FIGURES AND TABLES






