# Supplementary material for: Integrated quantitative proteomic and transcriptomic analysis of lung tumor and control tissue: a lung cancer showcase
Source: Oncotarget. 2016 Feb 22;7(12):14857–70. doi: 10.18632/oncotarget.7562 (PMC4924757; doi:10.18632/oncotarget.7562)
Supplement: Supplementary file 1 [file oncotarget-07-14857-s001.pdf]

Integrated quantitative proteomic and transcriptomic analysis of lung tumor and control tissue: a lung cancer showcase

Supplementary Materials

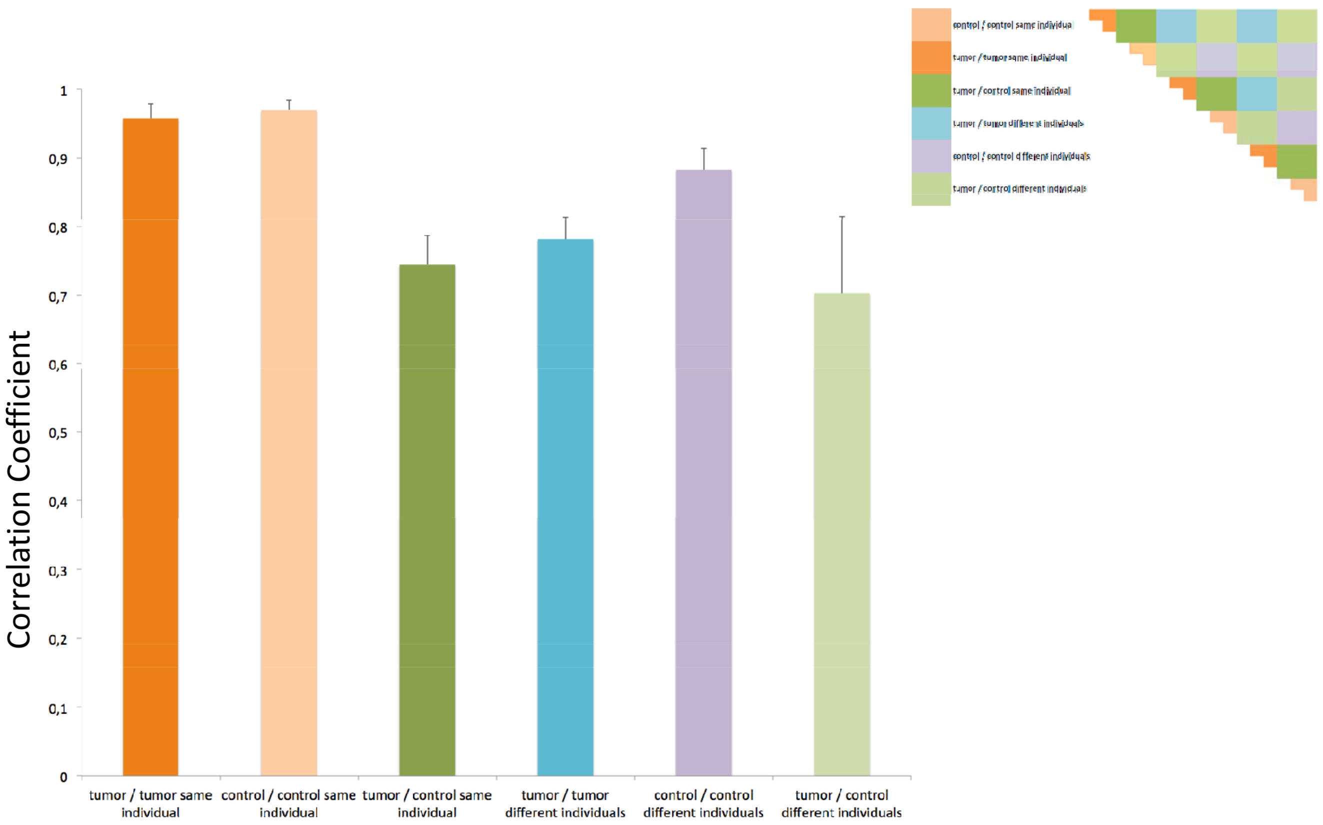

Supplementary Figure S1: Average correlation of the different groups of technical and biological replicates.

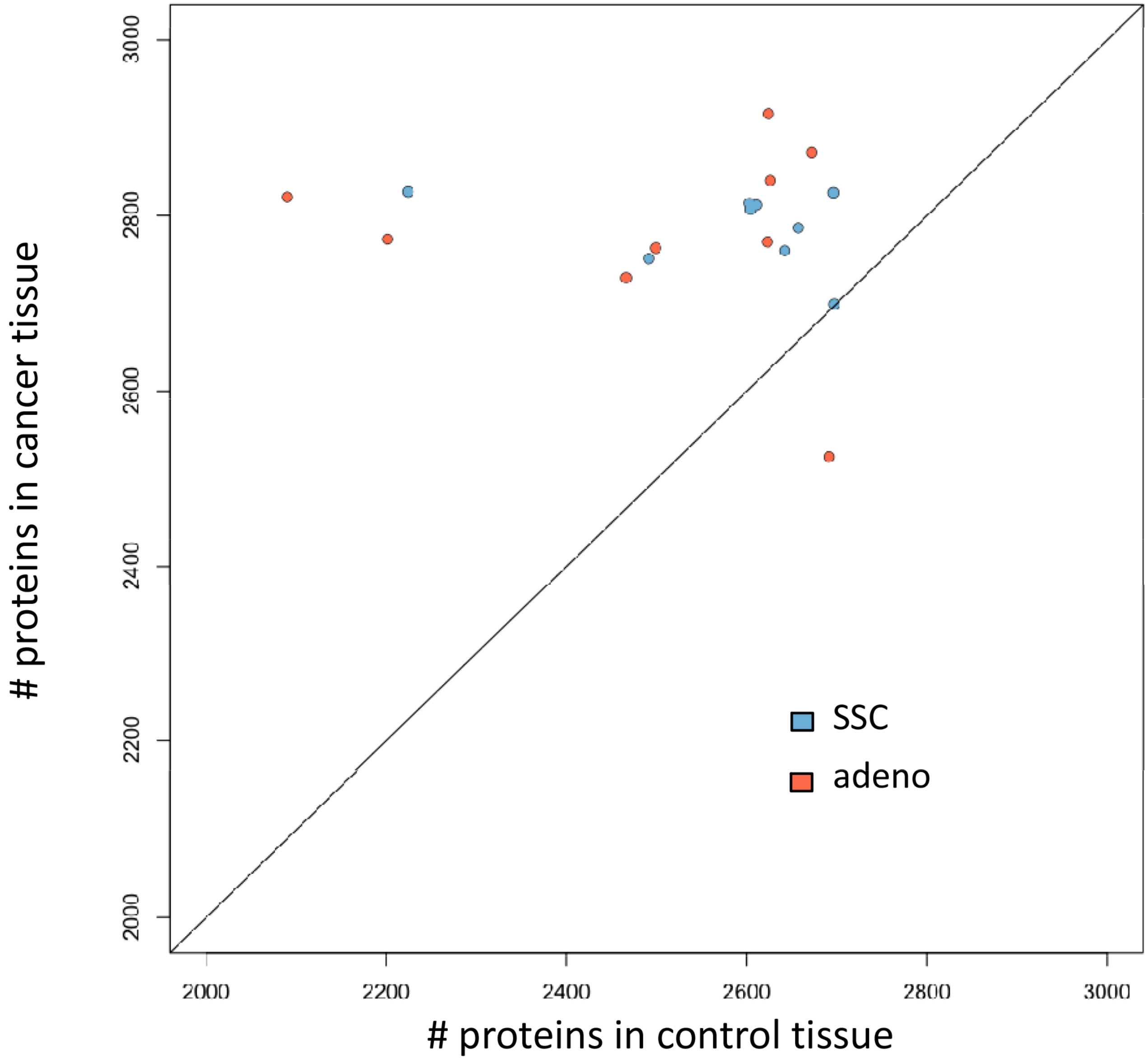

Supplementary Figure S2: Average number of proteins in cancer versus paired control tissue. In 16/18 cases lung cancer samples had increased protein repertoire, in one case both numbers matched and in one case the control tissue had a larger number of proteins detected by MS.

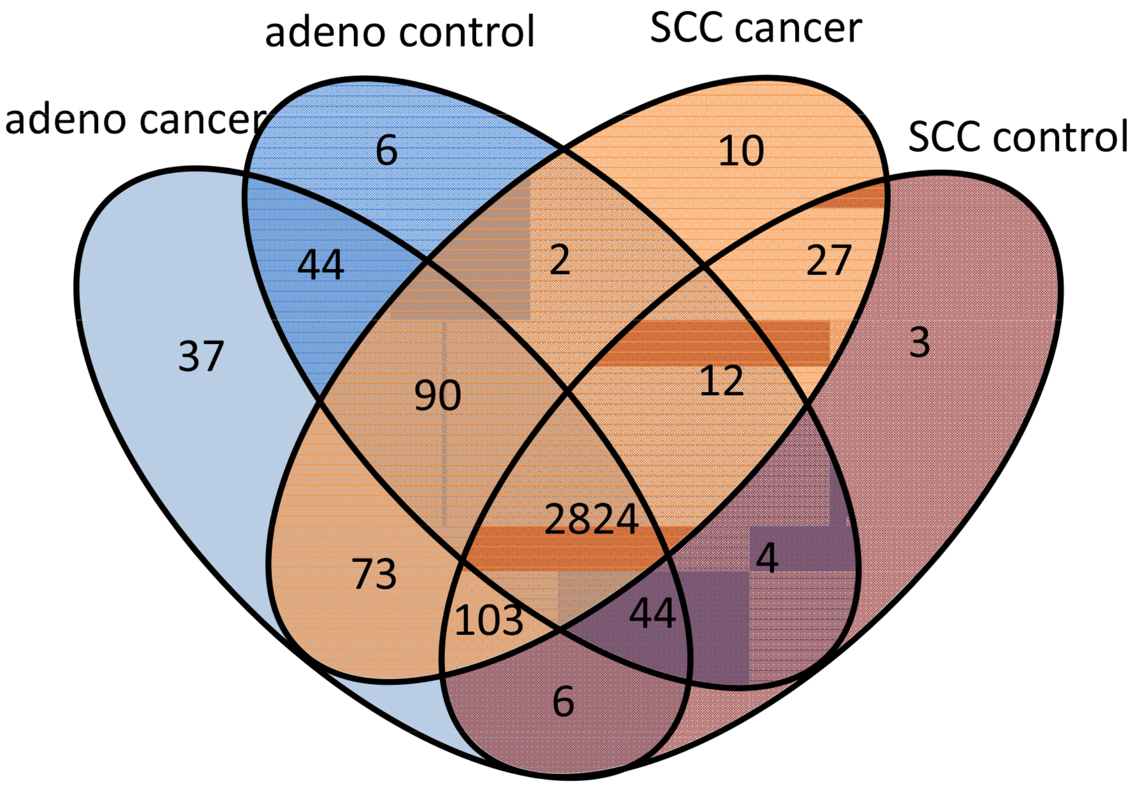

Supplementary Figure S3: Venn diagram showing the number of proteins in the tumor and control tissues.

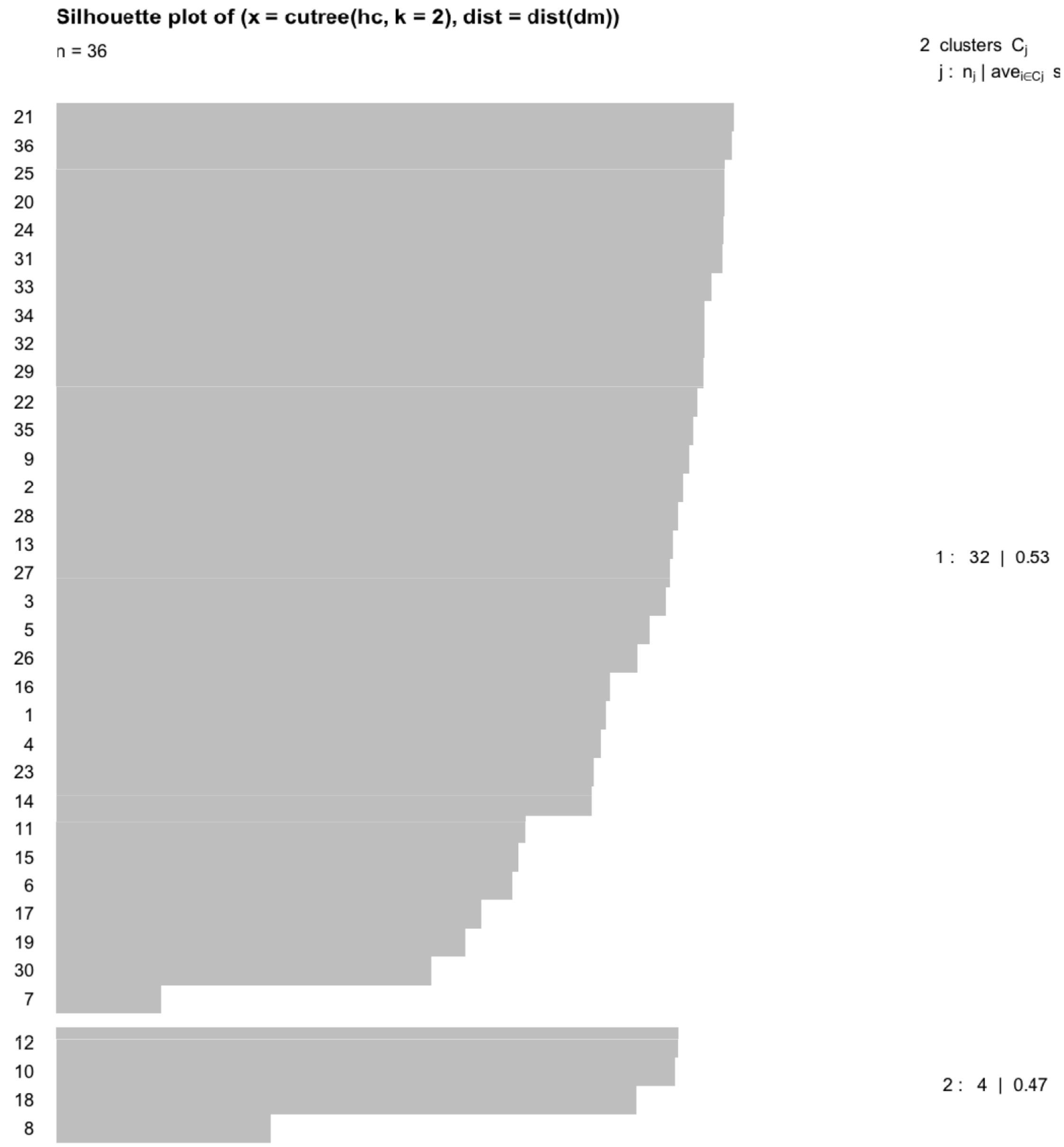

Supplementary Figure S4: Silhouette Scores for the 36 samples in 2 clusters.
